# Factors Affecting Adherence With Follow-up Appointments in HIV Patients

**DOI:** 10.7759/cureus.29424

**Published:** 2022-09-21

**Authors:** Katie A O'Connell, Shaheer Sherani, Alice Kisteneff, Karthik Bhat, Jarrett Slater, Christian F Klein, Brent Lavey, Ashlee Malone, Rehan Qayyum, Catherine J Derber

**Affiliations:** 1 Department of Internal Medicine, Division of Infectious Diseases, Eastern Virginia Medical School, Norfolk, USA; 2 Department of Medicine, Vanderbilt University Medical Center, Nashville, USA; 3 Department of Radiology, University of Virginia, Charlottesville, USA; 4 Vascular Surgery, Emory University School of Medicine, Atlanta, USA; 5 Department of Internal Medicine, University of Washington, Seattle, USA; 6 Emergency Medicine, The University of Tennessee Health Science Center, Memphis, USA; 7 Department of Internal Medicine, Eastern Virginia Medical School, Norfolk, USA

**Keywords:** follow-up appointment, uncontrolled hiv, hiv viral load, adherence to therapy, hiv care

## Abstract

Currently, the majority of new human immunodeficiency virus (HIV) infections are transmitted by individuals with untreated HIV. In this retrospective study, we examined associations between demographic factors, viral suppression, acquired immunodeficiency syndrome (AIDS) status (CD4 count <200), and adherence to clinical follow-up in individuals living with HIV. Of the 489 patients, 135 (27.6%) were females, 235 (48.1%) were over 50 years old, 191 (39.1%) had Medicaid, Medicare, or Ryan White Insurance, 25 (5.1%) had CD4 counts below 200, and 207 (42.3%) were adherent to their clinic appointments. In univariable logistic regression analysis, age and viral load detectability were significantly associated with patient adherence to their clinic appointment. In multivariable analysis, only age remained significantly associated with clinic appointment adherence (Odds Ratio=2.1; 95% Confidence Interval=1.4, 3.1; P<0.001). Patients 50 years old or younger were half as likely to be adherent to their clinic appointments than patients over 50 years old. Gender and insurance status were not associated with viral suppression or AIDS status. The results illustrate the need for increased age-specific outreach to improve clinical adherence in younger individuals.

## Introduction

The US Department of Health & Human Services' (HHS') ending the HIV epidemic Plan for America intends to increase the human immunodeficiency virus (HIV) viral suppression rate from an estimated 53% in 2020 to 90% by 2030 [1]. In addition, HHS plans to establish additional and expand existing treatment programs to both increase adherence to antiretroviral therapy (ART) and reduce 80% of annual new infections transmitted by individuals with untreated HIV [1]. Successful implementation of this plan requires a thorough understanding of local community demographics and treatment barriers. A lower viral load suppression rate has been observed more commonly in Black and Hispanic populations relative to white populations, which may reflect healthcare disparities and social determinants of health [2].

The last decade has seen significant advances in HIV prevention and treatment such as Undetectable=Untransmittable (U=U), pre-exposure prophylaxis, and effective single tablet regimens [3-5]. Historically, high pill burden and increased drug toxicity have been associated with low treatment adherence, and recent single tablet regimens may minimize these factors [6]. Although the effect of demographics on treatment adherence in HIV patients has been explored previously, there is less information about how these advances in treatment have impacted the relationship between demographics and adherence patterns. This study describes the factors impacting HIV ART adherence in a southern metropolitan area. Using CD4+ count and viral load as surrogates for ART adherence in a retrospective study, we evaluated the association of viral suppression rates with age, gender, insurance status, and number of infectious disease appointments within a two-year period.

## Materials and methods

The study was conducted at the HIV clinic of a tertiary care academic medical center. The center has a Ryan White program and is a referral source for a population of 1.8 million in the region. The study protocol and methods were approved by the Institutional Review Board (#18-03-FB-0044). Clinical and demographic data were collected retrospectively from patients who were seen at this HIV clinic during a two-year period (January 1, 2017, through December 31, 2018), were between the ages of 18 and 89 years at the time of data retrieval (July 2020), and who were prescribed ART for at least six months. Patients were excluded if they were on ART for less than six months or if there was no information to determine their adherence to ART. ART adherence was determined by viral load measurements: patients were categorized as adherent if the viral load was <200 viral copies/mL or non-adherent if the viral load was ≥200 copies/mL. To determine the severity of the disease, patients were further categorized as those who had AIDS (CD4 count <200 cells/μl) and those who did not have AIDS (CD4 count ≥200 cells/μl). Only the most recent laboratory values were used for this study. Patients were categorized into two age groups: those 50 years of age or younger and those who were older than 50 years. Insurance status was categorized into public (Medicaid, Medicare, or Ryan White) or other (such as private insurance). Adherence to follow-up was defined as being seen in the HIV clinic every six months. Gender and age were used as reported in the medical records.

Data were summarized as mean (standard deviation), median (interquartile range), and percentages as appropriate. The student’s t-test, chi-square test, or Fischer's exact test were used as appropriate to examine the relationship between adherence to ART and demographic or clinical variables. Univariable logistic regression was used to examine the effect size of each clinical and demographic variable on adherence to ART. Subsequently, we developed a single multivariable logistic regression model that includes all demographic and clinical variables to determine effect sizes in the presence of all variables. All analyses were conducted in Stata 16.1 (College Station, Texas), and a P<0.05 was considered significant.

## Results

Of the 489 patients included in the study, 135 (27.6%) were females, 235 (48.1%) were older than 50 years, 191 (39.1%) had Medicaid, Medicare, or Ryan White Insurance, 25 (5.1%) had CD4 count <200, and adherence to the appointment was noted in 207 (42.3%). The mean (standard deviation) age of the participants was 47.9 (13.4) years and the CD4 count was 744 (362) cells/μl. Viral load was <200 copies/mL in 428 (87.5%) patients, 202 (41.3%) patients had non-detectable viral load, and the median (interquartile range) of detectable viral load was 66 (400) copies/mL (Table 1).

**Table 1:** Study Population Characteristics Data are presented as mean (standard deviation) unless noted otherwise. P-values were calculated using the student’s t-test, Kruskal-Willis test, or chi-square test as appropriate. Abbreviations: N = number; CI = confidence interval; IQR= interquartile range

Variable	Appointment Adherence (N=207)	Appointment Non-Adherence (N=282)	Odds Ratio (95%CI); P-value
Age, years	51.1 (12.7)	45.6 (13.4)	1.03 (1.02, 1.05); <0.0001
Age>50 years, N (%)	121 (58.4)	114 (40.4)	2.07 (1.44, 2.99); <0.0001
Females, N (%)	66 (31.9)	69 (21.6)	1.44 (0.96, 2.14); 0.07
CD4 Count, cells/uL	769 (351)	726 (370)	1.00 (1.001, 0.999); 0.19
CD4 Count <200 cells/uL, N (%)	6 (2.9)	19 (6.7)	0.41 (0.16, 1.05); 0.06
Treatment Adherence, N (%)	189 (91.3)	239 (84.7)	1.89 (1.05, 3.38); 0.03
Viral Load Detectable, N (%)	110 (53.1)	177 (62.8)	0.67 (0.47, 0.97); 0.03
Viral Load (copies in 100s/uL), median (IQR) only when detectable	55 (54)	75 (3261)	0.996 (0.994, 0.999); 0.01
Medicaid, Medicare, or Ryan White	76 (36.7)	115 (40.8)	1.19 (0.82, 1.72); 0.36

In univariable logistic regression analysis, age and viral load detectability were significantly associated with patient adherence to their clinic appointment (Table 1). Each one-year increase in age was associated with a 3% higher odds of clinic appointment adherence (Odds Ratio (OR)=1.03; 95% Confidence Interval (CI) = 1.02, 1.05; P<0.0001). Similarly, patients older than 50 years were twice as likely to be adherent to their clinic appointments (OR=2.07; 95%CI = 1.44, 2.99; P<0.0001). On the other hand, patients with a detectable HIV viral load had 33% lower odds of being adherent to their appointment (OR=0.67; 95%CI = 0.47, 0.97; P=0.03). Among patients in whom viral load was detectable, every 100 copies/µL increase in viral load was associated with 0.1% lower odds of adherence to clinic appointments (0.994, 0.999; 0.01). However, in multivariable analysis, only age remained statistically significantly associated with clinical appointment adherence, and viral load was no longer associated with adherence (Table 2).

**Table 2:** Results of Multivariable Analysis Abbreviations: CI = confidence interval

Variable	Categories	OR (95%CI); P-value
Gender	Male	Reference
	Female	1.39 (0.91, 2.10); 0.12
Age	50 years or younger	Reference
	>50 years	2.09 (1.42, 3.07); <0.001
Viral Load	Not detectable	Reference
	Detectable	0.70 (0.47, 1.04); 0.08
CD4 Count	>200 cells/uL	Reference
	≤200 cells/uL	0.41 (0.14, 1.14); 0.09
Treatment Adherence	Adherent to treatment	Reference
	Not Adherent to treatment	0.82 (0.44, 1.53); 0.54
Insurance	Medicare, Medicaid, Ryan-White	Reference
	Private Insurance	1.38 (0.93, 2.05); 0.11

## Discussion

In this study, we found that age was an independent predictor of adherence to follow-up in HIV patients: older patients are more likely to adhere to their follow-up appointments than younger patients. Although viral load was also a factor in univariable analysis, it was no longer significant after adjustment for other variables. Adherence to clinical follow-up is important in the management of HIV, as is demonstrated by current guidelines. According to current guidelines, “retention in care” is defined as at least one medical visit every six months over the past two years [7]. The HIV/AIDS Bureau Ryan White & Global HIV/AIDS programs include this medical visit frequency as a core performance measure [8]. The importance of medical visit adherence is highlighted as a necessary precursor to achieving viral load suppression in the HIV Care Continuum [9]. This study reports that age is an important and independent factor associated with adherence to medical visits.

Potential causes for poor adherence to medical visits in younger HIV patients may include competing demands with work and childcare, lack of other medical co-morbidities, or symptoms that might drive seeking medical care, lack of transportation, substance use, and untreated mental health disease. Alternatively, survival bias may be a reason for the finding that older individuals were more likely to adhere to medical visit appointments. In other words, those who were not adherent to medical visits may not live long enough to be older - the proportion of HIV-related deaths was 48.6% among HIV patients aged 13-24 years and 30.0% among those aged ≥55 years [10].

Importantly, decreased adherence to clinic visits does not necessarily mean a lack of viral load suppression. In the most recent “Prevalence-based HIV Care Continuum” for the United States, although only 50% of people diagnosed with HIV were retained in care, 57% of all individuals with HIV achieved viral load suppression [9]. Our clinic population had an overall viral load suppression rate of 87.5%, despite a relatively low clinic visit adherence of 42.3%. Although lack of adherence to medical clinic visits was associated with higher viral loads in the unadjusted logistic regression analyses, this was not statistically significant in the adjusted analysis.

Historically, studies have cited an increased loss of follow-up in younger patients with inadequate medication adherence in young people [11-18]. However, it is important to note that a number of these studies were done over 10 years ago, before campaigns such as “Undetectable=Untransmittable” (U=U), in early 2016, and effective single-tablet regimens [5]. Despite this campaign and significant advances in HIV care, our study shows some of these gaps in adherence to medical visits remain. Future strategies should aim to engage younger populations in care. In addition to improved viral load suppression, regular medical visits provide an opportunity to address the risk for sexually transmitted infections, substance use interventions, and potential barriers to care.

Our study had several limitations, including the use of a single institution site and a lack of data related to behavioral and/or medical comorbidities. Additionally, appointment adherence was classified dichotomously and the frequency of visits was not recorded as a continuous variable. We did not confirm whether patients were actually scheduled every six months and if so, whether the missed appointment reflected a cancellation by the clinic staff or other factors beyond the patient’s control. Measures of prescription adherence and effectiveness such as pick up of medication from the pharmacy and viral drug resistance were also not assessed. Moreover, younger populations may be recently diagnosed and therefore were expected to have a detectable viral load. However, in this study, individuals were only selected if they had been prescribed ART for six months, at which point, their viral loads are likely to be undetectable, reducing the effect of the aforementioned limitation [19]. Additionally, residual confounding remains possible, as we could not capture all potential confounding factors such as other medical comorbidities, medications, mental health, and substance use. Lastly, individuals were not tracked for movement out of the region or incarceration and therefore may have been followed at a different clinic and were misclassified in this study. Of note, this study took place before the COVID pandemic; the COVID-19 pandemic will likely add another dimension to measuring gaps in adherence to medical visits and medication adherence.

## Conclusions

In summary, our study identified younger individuals to be less likely to adhere to HIV follow-up appointments in comparison to their older counterparts. To meet the goals outlined in HHS’ Ending the HIV Epidemic, efforts to increase the engagement of young people in HIV care should be explored. Our study validates previous literature suggesting that clinical visit adherence is higher in older patients living with HIV. However, it also highlights that while retention in care is inversely associated with viral load suppression, a decrease in regular medical follow-up visits does not necessarily mean poor viremic control. Additional studies are needed to determine what factors lead both to a medical visit and medication adherence and to further explore the relationship between adherence to medical clinic visits and HIV viral load. Our study findings also raise important clinical implications for our current standards for retention in care, primarily further exploring whether some individuals with sustained viral load suppression on antiretrovirals may benefit from a decreased medical visit frequency.
